# Fifty-Year Fate and Impact of General Medical Journals

**DOI:** 10.1371/journal.pone.0012531

**Published:** 2010-09-01

**Authors:** John P. A. Ioannidis, Lazaros Belbasis, Evangelos Evangelou

**Affiliations:** 1 Department of Hygiene and Epidemiology, University of Ioannina Medical School, Ioannina, Greece; 2 Biomedical Research Institute, Foundation for Research and Technology-Hellas, Ioannina, Greece; 3 Department of Medicine, Tufts Clinical and Translational Science Institute and Institute for Clinical Research and Health Policy Studies, Tufts Medical Center, Tufts University School of Medicine, Boston, Massachusetts, United States of America; 4 Department of Epidemiology, Harvard School of Public Health, Boston, Massachusetts, United States of America; University of Pittsburgh Medical Center, United States of America

## Abstract

**Background:**

Influential medical journals shape medical science and practice and their prestige is usually appraised by citation impact metrics, such as the journal impact factor. However, how permanent are medical journals and how stable is their impact over time?

**Methods and Results:**

We evaluated what happened to general medical journals that were publishing papers half a century ago, in 1959. Data were retrieved from ISI Web of Science for citations and PubMed (Journals function) for journal history. Of 27 eligible journals publishing in 1959, 4 have stopped circulation (including two of the most prestigious journals in 1959) and another 7 changed name between 1959 and 2009. Only 6 of these 27 journals have been published continuously with their initial name since they started circulation. The citation impact of papers published in 1959 gives a very different picture from the current journal impact factor; the correlation between the two is non-significant and very close to zero. Only 13 of the 5,223 papers published in 1959 received at least 5 citations in 2009.

**Conclusions:**

Journals are more permanent entities than single papers, but they are also subject to major change and their relative prominence can change markedly over time.

## Introduction

Medical journals shape clinical practice, health policy, public health, and biomedical research. Many people think of journals as stable values and don't contemplate that many influential journals may become non-influential or cease to exist in the future. One needs to examine the history of journals to understand these possibilities of change and decay. General medical journals emerged in the Age of Enlightenment. For example *Lancet* started in 1823 and *NEJM* in 1812, changing names several times (*New England Journal of Medicine and Surgery, New England Medical Review and Journal, Boston Medical and Surgical Journal*) before taking its current name in 1928. In the last decades, the number of journals has grown rapidly, including an increasing list of specialty venues [1]. Just as new journals appear, existing journals may change, or may stop their circulation. Moreover, the relative impact of specific journals compared to others changes over time. Journals' impact is traditionally measured by citation metrics, such as the Journal Impact Factor (JIF) [2] that takes into account the number of citations received by very recent papers. How does this compare with the impact of papers published by these same journals long ago?

Here we examine the long-term fate and impact of general medical journals. We have taken a snap shot of journals publishing papers 50 years ago, in 1959. We have examined the fate of these journals in the subsequent half-century and assessed the impact of the papers they published in 1959 as compared with the current JIF.

## Methods

We identified all journals that had published articles indexed in the General and Internal Medicine subject category by Thomson ISI Web of Science [3] for 1959. With appropriate subscription, it is possible to refine the searches in ISI so as to include specific subject categories. Then we searched the Journal function in PubMed to see if these venues continued or stopped publication, and if so, when. We also recorded whether journals merged with other journals, and if they changed names. We also traced previous names (before 1959) of each journal and when the journal (or its predecessors) had first been published.

We retrieved all items published in the eligible journals in 1959 and retained only those categorized by ISI as articles or reviews. The definition of what constitutes an article, a review or other item has been a contentious issue and some journals may try to increase their impact factor by publishing items that are not categorized as papers counted in the denominator of impact factor calculations. We have tried to avoid adding another layer of subjective tagging by re-characterizing the category of each of the published items ourselves, and thus we adopted the tagging provided already by ISI. However, this caveat should not be dismissed as it may affect the impact factor calculations of some journals.

We recorded from ISI the total citations received until the end of 2009 only for the articles and reviews of each journal (excluding citations to other items). We estimated the average 50-year citation impact per paper (article or review) for each journal as the ratio of the total citations (during 1959–2009) to articles and reviews published in 1959 divided by the number of such papers. Similarly, we estimated the average 2-year citation impact per paper as the ratio of the total citations during 1960–1961 to articles and reviews published in 1959 divided by the number of such papers. We assessed the Pearson correlation coefficient between the 50- and 2-year citation impact per paper and the latest JIF (Journal Citation Reports, 2008 edition). JIF uses in its calculation two years of recent publications and one year of citations (e.g. citations in 2009 for papers published in 2008 and 2007) and counts in the nominator also citations to items other than articles and reviews [2]. Therefore, we also estimated the 2-year citation impact per paper for papers published in 2007 for symmetry of definition to the respective 1959 metric and sensitivity analyses were also performed with the traditional definition of JIF with similar results (not shown in detail).

Furthermore, we have examined how many of the 20 general or internal medicine journals with the highest impact factors currently (per Journal Citation Reports 2008) were not published 50 years ago (neither with the same nor different name). Given that journals may be seen as businesses, for comparison, we also examined how many of the current top businesses worldwide (based on the Fortune 500 global edition, 2010), were not yet incorporated 50 years ago with information obtained from wikipedia.

Finally, we also assessed which articles published in 1959 received at least 5 citations in 2009.

Statistical analyses were performed in SPSS version 17.0 (SPSS Inc.). P-values are 2-tailed.

## Results

### Fate of journals

We identified 27 eligible journals in 1959 (Table 1). Four journals were no longer published by 2009. Two of them, the *Transactions of the Associations of American Physicians* and the *Bulletin of the Johns Hopkins Hospital* were among the oldest general journals and remained highly influential in medical research for a century (they had been launched in 1886 and 1889, respectively) before stopping circulation. Another 7 journals had changed names (two of those had also merged with other journals), but they still continued publication currently. One of them, the *Annales de l'Institut Pasteur* was considered as a journal covering the General and Internal Medicine category (in addition to Microbiology) in 1959, but lost this character in its subsequent transformation. Sixteen journals continued publication currently with the same name as in 1959.

**Table 1:** The fate of general and internal medicine journals publishing in 1959.

Journal	Start*	Name change by 1959*	Fate of journal after 1959
Acta Medica Scandinavica	1868	Yes	Continued as Journal of Internal Medicine after 1988
American Journal of Medicine	1946	No	Publishing with same name
American Journal of the Medical Sciences	1820	Yes	Publishing with same name
Annales de l' Institut Pasteur	1877	No	Continued with different names after 1972 (Annales de Microbiologie; Annales de l' Institut Pasteur. Microbiologie; Annales de l' Institut Pasteur. Microbiology; Research in Microbiology since 1989 until now)
Annals of Internal Medicine	1927**	Yes**	Publishing with same name
Annual Review of Medicine	1950	No	Publishing with same name
Archives of Internal Medicine	1908	Yes	Publishing with same name
Biken Journal	1958	No	Stopped publication in 1987
British Medical Bulletin	1943	No	Publishing with the same name
British Medical Journal	1840	Yes	Publishing with the same name
Bulletin of the Johns Hopkins Hospital	1889	No	Continued as the Johns Hopkins Medical Journal after 1966, stopped publication in 1982
Deutsche Medizinische Wochenschrift	1875	Yes	Publishing with the same name
Deutsches Archiv für Klinische Medizin	1865	No	Merged with Zeitschrift für Klinische Medizin and continued as Archiv für Klinische Medizin since 1966 which was then continued by the European Journal of Clinical Investigation since 1970
Harvey Lectures	1905	No	Publishing with the same name
JAMA	1848	Yes	Publishing with the same name
Journal of Laboratory and Clinical Medicine	1915	No	Continued as Translational Research: the Journal of Laboratory and Clinical Medicine after 2006
Klinische Wochenschrift	1864	Yes	Continued as Clinical Investigation after 1991, journal not possible to locate currently
Lancet	1823	No	Publishing with same name
Medical Clinics of North America	1917***	Yes***	Publishing with same name
Medicine (Baltimore)	1922	No	Publishing with same name
New England Journal of Medicine	1812	Yes	Publishing with same name
Presse Medicale	1893	No	Continued as Nouvelle Presse Medicale after 1971, then named again Presse Medicale since 1983
Proceedings of the Royal Society of Medicine-London	1809	Yes	Continued as Journal of the Royal Society of Medicine after 1977
Proceedings of the Staff Meetings of the Mayo Clinic	1926	No	Continued as Mayo Clinic Proceedings after 1963
Quarterly Journal of Medicine	1907	No	Continued as QJM after 1994
Transactions of the Association of >American Physicians	1886	No	Continued as Proceeding of the Association of American Physicians after 1993, stopped publication in 1999
Zeitschrift für Klinische Medizin	1879	No	Merged with Deutsches Archiv für Klinische Medizin and continued as Archiv für Klinische Medizin since 1965 which was then continued by the European Journal of Clinical Investigation since 1970

*considering also predecessor journals.

**the Annals of Internal Medicine were first published in 1927 but they succeeded the Annals of Clinical Medicine, for which we could not find the first publication year.

***the Medical Clinics of North America were first published in 1917 but they succeeded the Medical Clinics of Chicago for which we could not find the first publication year.

Eleven of the 27 journals had already succeeded a predecessor with a different name before 1959 (Table 1). Occasionally, a journal changed multiple names. For example, *BMJ* had started in 1840 with the *Provincial Medical Journal and Retrospect of the Medical Sciences*, which became the *Provincial Medical and Surgical Journal* in 1844–1952, then merged with the *London Journal of Medicine* (which had been published in 1849–1852) to become the *Association Medical Journal* in 1853–1856, finally renamed to *British Medical Journal* in 1857.

Of the 27 journals, only 6 were published continuously with the same name since their first circulation. Of these 6, only *Lancet* preceded the 20^th^ century.

### Citation impact of journals

In the 50-year frame, *Lancet* received the highest number of citations for the articles it published in 1959, followed at a distance by *NEJM, BMJ, JAMA*, and *American Journal of Medicine* that received approximately the same number of citations among them (Table 2).

**Table 2:** The impact of general and internal medicine journals publishing in 1959.

Journal	Papers (1959)	Citations Received in 1960–1961	Citations Received in 1959–2009	2-year Citations per Paper	50-year Citations per Paper	2008 JIF*	2-year Citations per Paper (2007)
Acta Medica Scandinavica	165	338	2909	2.05	17.63	5.412	10.09
American Journal of Medicine	185	1234	10889	6.67	58.86	5.105	6.78
American Journal of the Medical Sciences	154	389	2349	2.53	15.25	1.360	2.44
Annales de l' Institut Pasteur	160	239	1275	1.49	7.97	2.055	3.88
Annals of Internal Medicine	204	856	5784	4.20	28.35	17.457	28.65
Annual Review of Medicine	19	14	110	0.74	5.79	10.985	17.09
Archives of Internal Medicine	221	800	5579	3.62	25.24	9.110	17.69
Biken Journal	31	78	607	2.52	19.58	NP	NP
British Medical Bulletin	43	273	1210	6.35	28.14	3.277	4.84
British Medical Journal	475	1702	11137	3.58	23.45	12.827	11.30
Bulletin of the Johns Hopkins Hospital	35	136	2082	3.89	59.49	NP	NP
Deutsche Medizinische Wochenschrift	371	333	1500	0.90	4.04	0.625	1.02
Deutsches Archiv für Klinische Medizin	25	24	92	0.96	3.68	2.784	5.35
Harvey Lectures	9	1	23	0.11	2.56	NI	NI
JAMA	638	1874	11014	2.94	17.26	31.718	47.48
Journal of Laboratory and Clinical Medicine	234	767	6047	3.28	25.84	1.984	3.96
Klinische Wochenschrift	224	509	2293	2.27	10.24	NP	NP
Lancet	554	3285	19387	5.93	34.99	28.409	43.49
Medical Clinics of North America	111	144	1106	1.30	9.96	2.214	3.97
Medicine	15	144	1777	9.60	118.47	4.329	10.13
New England Journal of Medicine	420	1996	12572	4.75	29.93	50.017	73.73
Presse Medicale	522	237	1328	0.45	2.54	0.593	.81
Proceedings of the Royal Society of Medicine-London	236	171	1564	0.72	6.63	1.356	2.09
Proceedings of the Staff Meetings of the Mayo Clinic	94	181	964	1.93	10.26	4.811	8.01
Quarterly Journal of Medicine	30	172	2732	5.73	91.07	2.483	4.94
Transactions of the Association of American Physicians	28	98	727	3.50	25.96	NP	NP
Zeitschrift für Klinische Medizin	20	39	126	1.95	6.30	2.784	5.35

*Thomson ISI Journal Impact Factor, as derived from the 2008 Journal Citation Reports edition. It is calculated by dividing the total number of citations received in 2008 by items published in 2006 or 2007 by the number of original articles and reviews published in 2006 and 2007. Note that the nominator includes citations to all items published by a journal regardless of whether this is an original article, review or other type of item (e.g. editorial, essay, etc), while the denominator includes only original articles (articles and proceedings papers, a category that did not exist in 1959) and reviews. Moreover, the journal impact factor considers papers published during two years and the citations that they received in a single year (one or two years after their publication year, respectively). Therefore, the definition is slightly different from the 2-year citation impact metric that we used for papers published in 1959. The last column that shows the 2-year citation impact per paper for papers published in 2007 (citations to papers or reviews published in 2007 during 2008 and 2009 divided by the number of papers or reviews published in 2007) is conceptually identical to the respective metric for papers published in 1959. The Thomson ISI Journal Impact Factor was almost perfectly correlated with the 2-year impact of papers published in 2007 (r = 0.993, p<0.001) therefore correlations with the 1959-impact metrics were unaltered, when the 2-year impact of papers published in 2007 was used in the correlation analyses instead of the Thomson ISI Journal Impact Factor.

JIF: journal impact factor; NP: not publishing currently; NI: not indexed in ISI Web of Science currently.

*Medicine (Baltimore)* had the highest 50-year citation impact per paper. Each of the 15 papers that it published in 1959 received an average of 118 citations in 1959–2009. It was followed by the *QJM* (91 per paper), the *Bulletin of the Johns Hopkins Hospital* (59 per paper), the *American Journal of Medicine* (59 per paper), and *Lancet* (35 per paper), *NEJM* (30 per paper) and *Annals of Internal Medicine* (28 per paper). The 2-year impact per paper (1960–1961) was highly correlated with the 50-year impact per paper (r = 0.87, p<0.001).

The impact of the papers published in 1959 gives a very different picture compared with the current JIF of these journals. The correlation coefficient between the JIF and the 50-year (1959–2009) or 2-year (1960–1961) impact of these old papers is negligible (r = 0.04 [p = 0.86] and r = 0.25 [p = 0.26], respectively). Results remain the same, when journals without a current JIF are imputed as having JIF = 0 rather than excluded from the calculations (r = 0.05 [p = 0.81] and 0.27 [p = 0.17], respectively). Correlation estimates were practically identical when we used the 2-year impact for papers published in 2007 instead of the 2008 JIF (Table 2).

Overall, the 2-year impact per paper differed almost 100-fold across journals for papers published in 1959 (9.60 vs. 0.11) and similarly differed almost 100-fold across journals for papers published in 2007 (73.73 vs. 0.81). However, the absolute number of citations had increased 8-fold, given the much larger volume of the citing scientific literature in more recent years. Moreover, both the top-cited and worst-cited had changed over this half century (*Medicine* vs. *Harvey Lectures* in 1959; *NEJM* vs. *Presse Medicale* in 2007).

Three of the 5 journals with the highest 50-year impact have low or modest current JIF (<6) and another one has stopped circulation. The two journals with highest JIF currently (*NEJM* and *JAMA*) had far more modest citation impact based on the papers they published in 1959. Indicatively, none of the papers published by *NEJM* in 1959 received cumulatively more than 245 citations within 50-years, while 48 of the papers it published in 2006 received >245 citations just within 3 years from their publication.

### Top journals and top businesses

Of the 20 journals with the highest current impact factors in the “Medicine, general and internal” category, 5 were not even being published 50 years ago (PLoS Medicine launched in 2004, Cochrane Database of Systematic Reviews launched in 1994, American Journal of Preventive Medicine launched in 1985, Annals of Family Medicine launched in 2003, BMC Medicine launched in 2003). As a comparison, of the 20 top global companies currently, 6 had not yet been incorporated 50 years ago (Wal-Mart 1962, Japan Post Holdings 2007, Sinopec 2000, StateGrid 2002, China National Petroleum 1988, ING group 1991). Changes in names were very frequent both for top journals and for top companies (10 changed names in each group).

### Persistent citation of single papers

Only 226 of the 5,223 papers published in 1959 were cited at least once in 2009 and only 13 of them received at least 5 citations in 2009. All of them are classic papers in clinical investigation describing Prinzmetal's angina [4], the clinical significance of abnormal transaminases [5], treatment for obesity [6], fatal Asian influenza [7], chronic bronchitis [8], the first pharmacotherapy for depression with iproniazid [9], the association between behavior pattern and cardiovascular disease [10], treatment of menopause [11], pulmonary disease by atypical (anonymous) mycobacteria [12], internal mammary artery ligation [13], Turner syndrome [14], Down syndrome [15], and kuru spongiform encephalopathy [16].

## Discussion

Our evaluation shows that most of the influential journals of 50-years ago have survived to-date, but many have changed names and at least 4 have stopped circulation. The 50-year citation impact gives a very different picture about the relative influence of the general medical journal compared to the current JIF. If journals are seen as businesses, and one accepts that businesses come and go, then the changes in names and the lack of stability for journals is not much different to what in seen for top business corporations. Finally, less than 1 in 400 papers get 5 or more citations per year after 50 years have lapsed.

Few medical journals have kept their original name throughout their history. Sometimes, a change in name may be just a trivial modification, but in other occasions it may signify a change in course, focus, or perception about the mission of a journal, its audience and its content. Usually names of brands (not only journals) do not change unless there is a major reason, since the name of a brand is tied to the recognition and prestige of its products. Keeping abreast of developments in biomedical sciences is a perpetual challenge. Old disciplines disappear and new ones emerge. As an example of a disappearing discipline, until the advent of penicillin, the study and management of syphilis occupied a large specialty with many practitioners and scientists. Many scientific journals circulated with “syphilis” or related words in their names, including *(A. M. A.) Archives of Dermatology and Syphilology* (1920–1954); *American Journal of Syphilis, Gonorrhea, and Venereal Diseases* (1917–1954); *Annales de Dermatologie et de Syphiligraphie* (1868–1976); *Archiv für Dermatologie und Syphilis* (1889–1955); *Archives Belges de Dermatologie et de Syphiligraphie* (1938–1972); *The British Journal of Dermatology and Syphilis* (1917–1950); *Bulletin de la Société Française de Dermatologie et de Syphiligraphie* (1890–1976). All of these journals dropped syphilis from their names in the 1950s to 1970s – and some even ceased circulation. As an example of an emerging discipline, at least fourteen international journals are currently publishing with “proteomics” in their names (*Proteomics; Applied Genomics and Proteomics; Briefings in Functional Genomics & Proteomics; Cancer Genomics & Proteomics; Clinical Proteomics; Comparative Biochemistry and Physiology. Part D, Genomics & Proteomics; Current Proteomics; Expert Review of Proteomics; Genomics, Proteomics & Bioinformatics; Journal of Proteomics; Journal of Proteomics & Bioinformatics; Molecular & Cellular Proteomics; The Open Proteomics Journal; Proteomics. Clinical Applications*). None of them existed before 2001 when the first one (*Proteomics*) was launched.

While general journals are more stable than specialty journals by virtue of wider circulation or wider outreach and by their ability to accommodate material from whatever are the thriving or emerging specialties and disciplines *du jour*, they still have to struggle to survive themselves in a changing practice and research environment. Change and even decay are common. In fact, the first English general medical journal, the *Medicina Curiosa* that started in 1684, ceased its publication after only two issues [17]. The second one, the *Medical and Philosophical Commentaries*, which was launched in 1773 was very influential for almost two centuries [18], but changed names several times. It stopped circulation as *Edinburgh Medical Journal* in 1954.

JIF was first calculated [19] by Eugene Garfield in 1972 and has had an increasingly pervasive influence on appraising journals since then [2]. We should acknowledge that physicians and clinician-investigators did not depend on JIF or any other citation metrics to appraise the prestige of journals back in 1959 and it is difficult to say what they thought exactly about the relative ranking of journals back then. However, even as a retrospective exercise, the calculation of the impact metrics for papers published in 1959 shows the influence these papers had in the subsequent literature. Our analysis shows that the difference between the 50-year impact and current impact is not due to the short-term nature of calculations in estimating JIF. The relative impact of journals for their 1959-published papers was similar regardless of whether we examined 2- or 50-year citations. Apparently, sleeping beauties (articles that don't get cited initially, but receive many citations after several years) are rare [20]-[22].

Our data suggest that most influential articles were probably sent to different journals in 1959 than they would have gone to in 2009. *Medicine, QJM* and *American Journal of Medicine* were considered more prestigious than *NEJM* in 1959. Modest changes in the JIF ranking of journals have been seen in an analysis of 7 general journals covering a 12-year period (1994–2005) [23], but changes are more striking over half a century. It is difficult to see how and why perceptions about specific journals' ranking changed over time. Most likely this has been a very complex process and each journal has its own story to tell. General journals in these 50 years have had to compete for the coverage of an increasing number of specialties and sub-specialties that gradually became independent with their own stand-alone journals [24]. The long-tail distribution principle (few papers get a lot of citations, most papers get few citations) probably has operated during the whole length of this half century [25]. However, at a time when JIF was not yet proposed or at least not influential, leading papers were probably sent to diverse journals and a change in the relative citation impact of different journals could have happened both by wise editorial choices but also even by chance, given that it has been difficult to know with perfect certainty which papers will eventually be most influential [26], perhaps with the exception of some large landmark studies that are often collaborative and for which a large number of scientists awaits there results.

In the current environment where JIF has reached its apotheosis [27], it may be more difficult for a journal with substantially lower JIF to outperform one with much higher JIF in citation counts. Even if the lower-JIF journal publishes better papers, the community may feel obliged to cite papers published in journals with higher JIF [28], regardless of their merit. This preference leads to a spurious centralization of science to a few journals [29], [30], even though the extent of the centralization has been debated or even refuted [31]. It is unclear whether this situation will also continue in the future, as there is increasing interest to adopt additional, different metrics of impact [27], [32].

As for single papers, the vast majority of them have a citation life of anywhere between a few years to a couple of decades [2]. Few papers survive 50 years in the citation game. The citation decay is even more rapid in other scientific fields, such as molecular genetics, while some fields with slower turn-over (e.g. mathematics) may have more papers that continue to be cited for many years. Papers that are no longer cited have not necessarily been refuted and proven to be wrong in their inferences. Possibly most of them stop being cited simply because their field makes progress and investigators are inclined to cite papers that are more recent.

### Conclusion

Overall, our evaluation shows that single papers have a very transient presence in citations and with few exceptions they are rarely cited half a century after their publication. Journals are somewhat more permanent than single papers, but even most influential journals cannot avoid change and decay. The club of influential journals changes membership and ranking over time and in the long-term changes may become impressive. The very name of *journal*, derived from the old French *jurnal* and from the Latin *diurnus* denotes something which has a daily character, something that pertains to a single day, lasts for a day or is important for a day - only. The Greek equivalent word for journal is ΕΦΗΜΕΡΙΣ and not surprisingly it offers the root for what is *ephemeral*. As Heraclitus said, ΤΑ ΠΑΝΤΑ ΡΕΙ, everything changes.

## References

[1] Smith R (2006). The trouble with medical journals.. J R Soc Med.

[2] Garfield E (2006). The history and meaning of the journal impact factor.. JAMA.

[3] Reuters Thomson ISI Web of Science, accessed with subscription.

[4] Prinzmetal M, Kennamer R, Merliss R, Wada T, Bor N (1959). Angina pectoris. 1. A variant form of angina pectoris.. Am J Med.

[5] Wroblewski F (1959). The clinical significance of transaminase activities of serum.. Am J Med.

[6] Stunkard A, McLarenhume M (1959). The results of treatment for obesity – a review of the literature and report of a series.. Arch Intern Med.

[7] Martin CM, Kunin CM, Gottlieb LS, Barnes MW, Liu C (1959). Asian influenza-A in Boston, 1957-1958. I. Observations in 32 influenza-associated fatal cases.. Arch Intern Med.

[8] Fletcher CM, Elmes PC, Fairbairn AS, Wood CH (1959). The significance of respiratory symptoms and the diagnosis of chronic in a working population.. BMJ.

[9] West ED, Dally PJ (1959). Effects of iproniazid in depressive syndromes.. BMJ.

[10] Friedman M, Rosenman RH (1959). Association of specific overt behavior pattern with blood and cardiovascular findings – blood cholesterol level, blood clotting time, incidence of arcus senilis, and clinical coronary artery disease.. JAMA.

[11] Kupperman HS, Wetcheler BB, Blatt MHG (1959). Contemporary therapy of the menopausal syndrome.. JAMA.

[12] Runyon EH (1959). Anonymous mycobacteria in pulmonary disease.. Med Clinics N America.

[13] Cobb LA, Thomas GI, Dillard DH, Merendino KA, Bruce RA (1959). An evaluation of internal-mammary-artery ligation by a double-blind technic.. N Engl J Med.

[14] Ford CE, Jones KW, Polani PE, De Almeida JC, Briggs JH (1959). A sex-chromosome anomaly in a case of gonadal dysgenesis (Turner syndrome).. Lancet.

[15] Jacobs PA, Baikie AG, Brown WMC, Strong JA (1959). The somatic chromosomes in mongolism.. Lancet.

[16] Gajdusek DC, Zigas V (1959). Kuru-clinical, pathological, and epidemiological study of an acute progressive degenerative disease of the central nervous system among natives of the Eastern highlands in New-Guinea.. Am J Med.

[17] Colman E (1999). The first English medical journal, Medicina Curiosa.. Lancet.

[18] Chalmers I, Tröhler U (2000). Helping physicians to keep abreast of the medical literature: Medical and Philosophical Commentaries, 1773-1795.. Ann Intern Med.

[19] Garfield E (1972). Citation analysis as a tool in journal evaluation.. Science.

[20] Burrell QL (2005). Are “sleeping beauties” to be expected?. Scientometrics.

[21] Glanzel W, Garfield E (2004). The myth of delayed recognition.. Scientist.

[22] van Raan AFJ (2004). Sleeping beauties in science.. Scientometrics.

[23] Chew M, Villanueva EV, Van Der Weyden MB (2007). Life and times of the impact factor: retrospective analysis of trends for seven medical journals (1994-2005) and their Editors' views.. J R Soc Med.

[24] Rosvall M, Bergstrom CT (2010). Mapping change in large networks.. PLoS One.

[25] Michon F, Tummers M (2009). The dynamic interest in topics within the biomedical scientific community.. PLoS One.

[26] Young NS, Ioannidis JP, Al-Ubaydli O (2008). Why current publication practices may distort science.. PLoS Med.

[27] The impact factor game (2006). It is time to find a better way to assess the scientific literature.. PLoS Med.

[28] Perneger TV (2010). Citation analysis of identical consensus statements revealed journal-related bias.. J Clin Epidemiol.

[29] Evans JA (2008). Electronic publication and the narrowing of science and scholarship.. Science.

[30] Ioannidis JP (2006). Concentration of the most-cited papers in the scientific literature: analysis of journal ecosystems.. PLoS One 2006;.

[31] Lariviere V, Gingras Y, Archambault E (2009). The decline in the concentration of citations, 1990-2007.. J Am Soc Inf Sci Technol.

[32] Bollen J, Van de Sompel H, Hagberg A, Chute R (2009). A principal component analysis of 39 scientific impact measures.. PLoS One.

